# Possible Role of *HLA*-*G*, *LILRB1* and *KIR2DL4* Gene Polymorphisms in Spontaneous Miscarriage

**DOI:** 10.1007/s00005-016-0389-7

**Published:** 2016-03-14

**Authors:** Izabela Nowak, Andrzej Malinowski, Ewa Barcz, Jacek R. Wilczyński, Marta Wagner, Edyta Majorczyk, Hanna Motak-Pochrzęst, Małgorzata Banasik, Piotr Kuśnierczyk

**Affiliations:** 1 Laboratory of Immunogenetics and Tissue Immunology, Department of Clinical Immunology, Ludwik Hirszfeld Institute of Immunology and Experimental Therapy, Polish Academy of Sciences, Rudolfa Weigla 12, 53-114 Wroclaw, Poland; 2 Department of Surgical, Endoscopic and Oncologic Gynecology, Polish Mothers’ Memorial Hospital-Research Institute, Lodz, Poland; 3 First Chair and Clinic of Obstetrics and Gynecology, Medical University of Warsaw, Warsaw, Poland; 4 Department of Gynecology and Gynecologic Oncology, Polish Mothers’ Memorial Hospital-Research Institute, Lodz, Poland; 5 Faculty of Physical Education and Physiotherapy, Opole University of Technology, Opole, Poland; 6 Obstetric Gynecological Department, Disctrict Hospital Strzelce Opolskie, Strzelce Opolskie, Poland; 7 APC, Medical Analyses, Lodz, Poland

**Keywords:** HLA-G, LILRB1, Linkage disequilibrium, KIR2DL4, Spontaneous abortion

## Abstract

The KIR2DL4 receptor and its ligand HLA-G are considered important for fetal-maternal immune tolerance and successful pregnancy. The absence of a particular variant of KIR2DL4 might be a bad prognostic factor for pregnancy outcome. However, it could be compensated by the presence of the respective LILRB1 allele. Therefore, we investigated the *KIR2DL4*, *LILRB1* and *HLA*-*G* polymorphisms in 277 couples with spontaneous abortion and 219 control couples by HRM, PCR-SSP and RFLP methods. We found a protective effect of women’s heterozygosity in −716 *HLA*-*G* (*p* = 0.0206) and *LILRB1* (*p* = 0.0131) against spontaneous abortion. Surprisingly, we observed more 9A/10A genotypes of *KIR2DL4* gene carriers in the group of male partners from the miscarriage group in comparison to the men from the control group (*p* = 0.0288). Furthermore, there was no association of women’s *KIR2DL4* polymorphism with susceptibility to spontaneous abortion. Multivariate analysis indicated that women’s −716 *HLA*-*G* and *LILRB1* and men’s *KIR2DL4* 9A/10A are important in terms of the protection or susceptibility to miscarriage, respectively (*p* = 0.00968). In conclusion, a woman’s heterozygosity in *HLA*-*G* and *LILRB1* might be an advantage for a success of reproduction, but the partner’s heterozygosity in 9A/10A *KIR2DL4* alleles might not.

**Electronic supplementary material:**

The online version of this article (doi:10.1007/s00005-016-0389-7) contains supplementary material, which is available to authorized users.

## Introduction

Spontaneous abortion has been described as a condition where a multifactorial background plays a role in its etiology (Christiansen 2013; Diejomaoh 2015; Larsen et al. 2013; Sugiura-Ogasawara et al. 2014). The pathomechanism of spontaneous miscarriage is still not completely understood. Many scientists are searching for an explanation of this disease in immunological pathways, because the fetus is perceived by the mother as a semiallograft, which in normal pregnancy is not rejected (Medawar 1953). Many researchers are also looking for genetic biomarkers as a diagnostic tool. Among receptors which may have an influence on decidual-trophoblast cell interactions in pregnancy are KIR2DL4 (killer cell immunoglobulin-like receptor, two domains, long cytoplasmic tail, 4), LILRB1 (leukocyte immunoglobulin-like receptor, subfamily B, member 1) and their ligand, HLA-G (human leukocyte antigen G) (Li et al. 2009).

KIR2DL4 is unique among KIRs because of its structure, cellular localization and expression. This receptor possesses a single ITIM (immunoreceptor tyrosine-based inhibitory motif) in its cytoplasmic tail and a positively charged arginine in the transmembrane region. Therefore, it can potentially inhibit or activate cells functions. KIR2DL4 is expressed by natural killer (NK) CD56^bright^ cells and appears on the majority of decidual-placental NK cells but not on peripheral NK CD56^bright^ cells (Makrigiannakis et al. 2011; Rajagopalan and Long 2012).

The 9A allele of the *KIR2DL4* gene with the deletion of one adenine in exon 7 produces either a protein with a truncated cytoplasmic tail or one lacking the transmembrane region. This causes a lack of KIR2DL4 expression at the cell surface. In contrast, 10A alleles encode receptors that can be expressed at the cell surface (Goodridge et al. 2007, 2009). Hence, *KIR2DL4* polymorphism may have an influence on the interaction of decidual NK cells with HLA-G expressed on trophoblastic cells. Moreover, we could speculate that absence of KIR2DL4 could be compensated by the presence of a particular allele of LILRB1, also named ILT-2 (immunoglobulin-like transcript-2) (Li et al. 2009). *LILRB1* as well as *KIRs* was found in a gene cluster at chromosomal region 19q13.4. Multiple transcript variants encoding different isoforms have been found for this gene. The encoded protein contains four extracellular immunoglobulin domains, a transmembrane domain, and four cytoplasmic ITIMs (Martin et al. 2002). The receptor, which is expressed on immune cells (Apps et al. 2007) and placental stromal cells, primarily on fibroblasts and macrophages (McIntire et al. 2008), may bind to HLA-G and transduce a negative signal that inhibits stimulation of an immune response.

Even though HLA-G is classified as a non-classical class I HLA because of limited tissue distribution and lower polymorphism, 51 alleles of this molecule were identified (The IMGT database; October 2015). The most polymorphic sites of *HLA*-*G* were found in the promoter (positions −725, −716), in exon 8, and in 3′UTR (14 base pair insertion/deletion) of the gene, resulting in variation of HLA-G expression. The *HLA*-*G* gene, due to alternative splicing of its transcript, encodes seven proteins: four are bound with membrane (HLA-G1 to HLA-G4), while three (HLA-G5 to HLA-G7) appear as soluble proteins (Dahl and Hviid 2012; Donadi et al. 2011; Menier et al. 2010). In pregnancy, expression of HLA-G is determined by the kind of trophoblast and stage of pregnancy progression. HLA-G membrane-bound molecules (of maternal and paternal origin) are presented by all extravillous trophoblast subpopulations. Moreover, soluble isoforms (HLA-G5-7) were detected in maternal-fetal circulation, amniotic fluid, and all trophoblasts, including the syncytiotrophoblast, which is deprived of membrane-bound class I antigens (Dahl and Hviid 2012; McIntire et al. 2008).

In our case–control study we tested different genetic variants of *KIR2DL4* (9A/10A alleles and three intronic positions near this poly-adenine fragment), *LILRB1* (rs41308748 G>A) and *HLA*-*G* (−725 C>G>T, −716 T>G in the promoter region and a 14 base pair insertion/deletion in 3′UTR) in patients with spontaneous abortion and control women as well as in male partners of these two groups. To our knowledge, this is the first report concerning cumulative genotypes of *KIR2DL4*, *LILRB1* and *HLA*-*G* in couples and the possible association with miscarriage.

## Materials and Methods

### Study Design

All patients (and their partners) participating in the study were recruited from the Department of Surgical, Endoscopic and Oncologic Gynecology and Department of Gynecology and Gynecologic Oncology, Polish Mothers’ Memorial Hospital-Research Institute, Poland. Two hundred and seventy-seven couples, who had experienced spontaneous abortion (2–8 miscarriages) but were free from chromosomal aberrations, uterine anomalies, hormonal disturbances, and infections with *Toxoplasma*, *Chlamydia*, *Listeria*, and *Brucella*, were originally qualified for our study. Among them, 79 couples had two miscarriages (sporadic spontaneous abortion, SSA, with the mean age 32.08 ± 3.85 years; age range 25–41). The remaining group of 198 couples belonged to the recurrent spontaneous abortion group (RSA; with the mean age 32.78 ± 4.00 years; age range 24–46). These were selected on the basis of a history of three or more first trimester spontaneous abortion incidents with the same partner. Moreover, among the RSA group we selected 115 women (58.1 %) without autoantibodies. The remaining group of 83 RSA women (41.9 %) possessed a different set of autoantibodies, such as anticardiolipin, antinuclear, antithyroid, anti-β-glycoprotein, and factor LA. In the sporadic abortion group we could distinguish those possessing autoantibodies (35 women, 44.3 %) and without autoantibodies (40 women, 50.6 %). We had no data regarding autoantibodies concerning four women (5.1 %) from the SSA group. As we realized that our patient group was heterogeneous (in terms of presence of autoantibodies, factor V Leiden, antiphospholipid syndrome and mutations in *MTHFR 677C*>*T* and *1298A*>*C* positions), we decided to include all collected couples and use a multivariate analysis.

The control group was recruited from the 1st Department of Obstetrics and Gynecology, Medical University of Warsaw and from the Disctrict Hospital Strzelce Opolskie. This group consisted of 219 healthy couples with at least two healthy-born children and no history of miscarriage or endocrinological or immunological disorders: women with the mean age 32.29 ± 5.81 years, age range 22–68, and their partners with the mean age 33.97 ± 6.18 years, age range 25–70. Men from the spontaneous abortion group had a similar age to the men from the control group: mean age 34.2 ± 3.15 years, age range 27–41. Thus, both control and spontaneous abortion groups were age-matched. All tested individuals were of Polish origin. Experimental protocols were approved by the Local Ethics Committees (the agreement of Medical University of Wroclaw and Polish Mothers’ Memorial Hospital–Research Institute in Łódź) and informed consent was obtained from all individual participants included in the study.

### DNA Preparation and Genotyping

Genomic DNA was isolated from venous blood using the Invisorb Spin Blood Midi Kit (Invitek, Berlin, Germany) following the manufacturer’s instructions.

*KIR2DL4* 9A/10A alleles (rs11410751) and three other single nucleotide polymorphisms (SNPs) spanning the vicinity of the poly-adenine fragment, i.e. rs660773—position 9797 G>A (intron 7), rs660437—position 9769 C>A (intron 7), rs649216—9571 C>T (762), were distinguished by the high resolution melting (HRM) method and by restriction fragment length polymorphism (RFLP), respectively. Details of the genotyping have been described recently in details elsewhere (Nowak et al. 2015).

*HLA*-*G* genotyping in positions −725 C>G>T (rs1233334) and −716 T>G (rs2249863) was conducted by temperature gradient gel electrophoresis, and the 14 base pair insertion/deletion (rs66554220) of *HLA*-*G* was tested by the PCR-SSP (sequence-specific priming) method. Both methods have been described previously by Wiśniewski et al. (2010).

*MTHFR* *677C*>*T* and *MTHFR* *1298A*>*C* genotyping is described in Supplementary Material 1 and Supplementary Fig. 1–4.

*LILRB1* 5651G>A position (rs41308748, located in the 14th intron) genotyping is described in Supplementary Material 2 and Supplementary Figs. 5, 6. rs41308748 showed minor allele frequency (MAF) in controls (both women and men) MAF ≤ 0.09. To predict possible functional effects for this SNP we used the website: http://fastsnp.ibms.sinica.edu.tw (Yuan et al. 2006), which proposed it as the splicing site with the risk at the 3–4 level (with maximum 4).

The *LILRB1 5651* genotype distributions were deviated from Hardy–Weinberg equilibrium (HWE) (Tables 1, 2). Therefore, we sequenced 14 AA genotype samples (from all 22), seven samples for GA and five samples for GG genotype. We repeated digestion for 11 samples because of the suspicion of the partial digestion.

**Table 1:** Genotype frequencies in women group according to cases and controls

Polymorphism		Cases A		Cases B		Controls^a^		OR^b^	CI 95 %		Cases vs. controls
		*N*	%	*N*	%	*N*	%				
HLA-G 14 bp ins/del	del/del	27	34.2	73	36.9	74	33.8	1			*χ*_*df*=4_^2^ = 6.91 *p* = 0.1407
	del/ins	30	38	91	45.9	110	50.2	0.82	0.55	1.21	
	ins/ins	22	27.8	34	17.2	35	16	1.18	0.70	1.98	
	∑	79	100	198	100	219	100				
	H-W	*p* = 0.0415		*p* = 0.5551		*p* = 0.6754					
	*f*	0.237		0.044		–0.04					
HLA-G −725	CC	52	65.8	134	67.7	152	69.4	1			*χ*_*df=*6_^2^ = 4.69 *p* = 0.8051
	CG	19	24.0	50	25.3	57	26	0.99	0.66	1.49	
	CT	4	5.1	7	3.5	5	2.3	1.71	0.61	4.83	
	GG	4	5.1	6	3	5	2.3	1.56	0.54	4.48	
	GT	0	0	1	0.5	0	0	2.45	–	–	
	∑	79	100	198	100	219	100				
	H-W	*p* = 0.2833		*p* = 0.8097		*p* = 0.9989					
	*f*	0.049		0.014		0.019					
HLA-G −716	TT	23	29.1	51	25.8	58	26.5	1			*χ*_*df*=4_^2^ = 7.19 *p* = 0.1262
	GT	32	40.5	102	51.5	122	55.7	0.86	0.57	1.31	
	GG	24	30.4	45	22.7	39	17.8	1.38	0.82	2.32	
	∑	79	100	198	100	219	100				
	H-W	*p* = 0.1141		*p* = 0.7759		*p* = 0.0784					
	*f*	0.189		–0.031		–0.122					
KIR2DL4 9620	9A/9A	22	27.8	52	26.3	66	30.1	1			*χ*_*df*=4_^2^ = 0.86 *p* = 0.9302
	9A/10A	38	48.1	100	50.5	103	47	1.19	0.79	1.81	
	10A/10A	19	24.1	46	23.2	50	22.8	1.16	0.71	1.90	
	∑	79	100	198	100	219	100				
	H-W	*p* = 0.8218		*p* = 0.8896		*p* = 0.4174					
	*f*	0.036		–0.011		0.054					
KIR2DL4 9571	TT	23	29.1	55	27.8	71	32.4	1			*χ*_*df*=4_^2^ = 1.19 *p* = 0.8797
	CT	38	48.1	100	50.5	103	47	1.22	0.81	1.84	
	CC	18	22.8	43	21.7	45	20.5	1.23	0.75	2.03	
	∑	79	100	198	100	219	100				
	H-W	*p* = 0.8216		*p* = 0.8874		*p* = 0.4952					
	*f*	0.034		–0.014		0.046					
KIR2DL4 9769	CC	53	67.1	135	68.2	147	67.1	1			*χ*_*df*=4_^2^ = 6.53 *p* = 0.1589
	CA	23	29.1	63	31.8	66	30.1	1.02	0.69	1.50	
	AA	3	3.8	0	0	6	2.7	0.42	0.11	1.57	
	∑	79	100	198	100	219	100				
	H-W	*p* = 0.7153		*p* = 0.0056		*p* = 0.8187					
	*f*	0.0286		–0.189		–0.029					
KIR2DL4 9797	GG	23	29.1	55	27.8	70	32	1			*χ*_*df*=4_^2^ = 0.97 *p* = 0.9131
	GA	38	48.1	100	50.5	103	47	1.20	0.80	1.81	
	AA	18	22.8	43	21.7	46	21	1.19	0.72	1.96	
	∑	79	100	198	100	219	100				
	H-W	*p* = 0.8216		*p* = 0.8874		*p* = 0.4948					
	*f*	0.034		–0.014		0.048					
LILRB1 5651	GG	69	87.3	172	86.9	182	83.1	1			*χ*_*df*=4_^2^ = 4.28 *p* = 0.3688
	GA	10	12.7	22	11.1	35	16	0.69	0.41	1.16	
	AA	0	0	4	2	2	0.9	1.36	0.29	6.46	
	∑	79	100	198	100	219	100				
	H-W	*p* = 0.5481		*p* = 0.0159		*p* = 0.6806					
	*f*	–0.067		0.207		0.015					

*Cases A* patients with two miscarriages, *Cases B* patients with three or more miscarriages, *N* number of cases, *P* probability, *OR* odds ratio, *95* *% CI* confidence interval, *H*–*W* Hardy–Weinberg equilibrium, *f* < 0 corresponds to deficiency of homozygotes, *f* > 0 corresponds to excess of homozygotes, *f* = 0 in case of H–W

^a^Frequencies of *KIR2DL4* polymorphisms in controls were described previously by Nowak et al. (2015)

^b^OR computed for [cases A plus cases B] vs. controls

**Table 2:** Genotype frequencies in men group according to cases and controls

Polymorphism		Cases A		Cases B		Controls^a^		OR^b^	CI 95 %		Cases vs. controls
		N	%	N	%	N	%				
HLA-G 14 bp ins/del	del/del	29	36.7	61	30.8	75	34.2	1			*χ*_*df*=4_^2^ = 1.57 *p* = 0.8136
	del/ins	30	38	81	40.9	91	41.6	1.02	0.67	1.53	
	ins/ins	20	25.3	56	28.3	53	24.2	1.19	0.75	1.90	
	∑	79	100	198	100	219	100				
	H-W	*p* = 0.0418		*p* = 0.0107		*p* = 0.02					
	*f*	0.23		0.18		0.16					
HLA-G −725	CC	43	54.5	142	71.7	145	66.2	1			*χ*_*df*=6_^2^ = 11.1 *p* = 0.1959
	CG	31	39.2	46	23.3	55	25.1	1.10	0.73	1.65	
	CT	3	3.8	6	3	12	5.5	0.60	0.25	1.42	
	GG	2	2.5	3	1.5	5	2.3	0.78	0.24	2.61	
	GT	0	0	1	0.5	2	0.9	0.47	–	–	
	∑	79	100	198	100	219	100				
	H-W	*p* = 0.5701		*p* = 0.9154		*p* = 0.9989					
	*f*	–0.09		–0.008		0.008					
HLA-G −716	TT	28	35.4	42	21.2	52	23.7	1			*χ*_*df*=4_^2^ = 7.76 *p* = 0.1008
	GT	27	34.2	95	48	105	47.9	0.86	0.56	1.35	
	GG	24	30.4	61	30.8	62	28.3	1.02	0.63	1.65	
	∑	79	100	198	100	219	100				
	H-W	*p* = 0.0064		*p* = 0.6681		*p* = 0.5885					
	*f*	0.314		0.031		0.039					
KIR2DL4 9620	9A/9A	22	27.8	59	29.8	74	33.8	1			*χ*_*df*=4_^2^ = 4.12 *p* = 0.3904
	9A/10A	42	53.2	103	52	95	43.4	1.39	0.93	2.09	
	10A/10A	15	19	36	18.2	50	22.8	0.93	0.57	1.54	
	∑	79	100	198	100	219	100				
	H-W	*p* = 0.651		*p* = 0.4747		*p* = 0.0756					
	*f*	–0.072		–0.055		0.122					
KIR2DL4 9571	TT	22	27.8	59	29.8	78	35.6	1			*χ* ^2^ = 4.1 *p* = 0.3921
	CT	42	53.2	103	52	95	43.4	1.47	0.98	2.20	
	CC	15	19	36	18.2	46	21	1.07	0.64	1.76	
	∑	79	100	198	100	219	100				
	H-W	*p* = 0.651		*p* = 0.4747		*p* = 0.0979					
	*f*	–0.072		–0.055		0.113					
KIR2DL4 9769	CC	53	67.1	144	72.7	170	77.6	1			*χ*_*df*=4_^2^ = 4.07 *p* = 0.4033
	CA	23	29.1	45	22.7	42	19.2	1.39	0.90	2.15	
	AA	3	3.8	9	4.6	7	3.2	1.44	0.57	3.64	
	∑	79	100	198	100	219	100				
	H-W	*p* = 0.7153		*p* = 0.0568		*p* = 0.0597					
	*f*	0.028		0.15		0.14					
KIR2DL4 9797	GG	22	27.8	56	28.3	77	35.2	1			*χ*_*df*=4_^2^ = 4.6 *p* = 0.3304
	GA	42	53.2	107	54	97	44.3	1.51	1.01	2.27	
	AA	15	19	35	17.7	45	20.5	1.10	0.66	1.82	
	∑	79	100	198	100	219	100				
	H-W	*p* = 0.651		*p* = 0.2495		*p* = 0.168					
	*f*	–0.072		–0.093		0.095					
LILRB1 5651	GG	66	83.5	162	81.8	190	86.7	1			*χ*_*df*=4_^2^ = 2.24 *p* = 0.6987
	GA	10	12.7	28	14.1	24	11	1.31	0.76	2.25	
	AA	3	3.8	8	4.1	5	2.3	1.74	0.62	4.90	
	∑	79	100	198	100	219	100				
	H-W	*p* = 0.026		*p* = 0.0006		*p* = 0.0046					
	*f*	0.3		0.28		0.235					

*Cases A* patients with two miscarriages; *Cases B* patients with three or more miscarriages, *N* number of cases, *P* probability, *OR* odds ratio, *95* *% CI* confidence interval, *H*-*W* Hardy–Weinberg equilibrium, *f* < 0 correspond to deficiency of homozygotes, *f* > 0 correspond to excess of homozygotes, *f* = 0 in case of H-W

^a^ Frequencies of *KIR2DL4* polymorphisms in Controls were described previously by Nowak et al. (2015). ^b^ Odds ratio computed for [cases A plus cases B] vs. controls

### Statistical Analysis

Chi-square, *χ* ^2^, test was used to test the hypothesis that two groups have the same the distribution of genotype counts. When the sample sizes were small, distributions of the test statistics were estimated numerically. Odds ratio (OR) and confidence interval for them at 1 − *α* = 0.95 were computed as the measures of effect size. When it was reasonable, we assumed log additive model of association between genotype and risk of miscarriages. Genetic differences between cases (*Y* = 1) and controls (*Y* = 0) were tested with model $h\left[ {P\left( {Y = 1|\underline{x} } \right)} \right] = \alpha + \underline{\beta }^{T} \underline{x}$, where $\underline{x}$ is matrix of genetic predictors and *h* is logit. Number of miscarriages, *k*, among cases was investigated with model defined as $h\left[ {P\left( {Y \le k|\underline{x} } \right)} \right] = \alpha_{k} + \underline{\beta }^{T} \underline{x}$. Results were adjusted to age, autoantibodies and *MTHFR* polymorphisms. When necessary, coefficients *α*, *β* and their standard errors were estimating with bootstrap sampling (*B* = 4999). To summarize predictive power of the model we used a measure of proportional reduction in sum of squared errors (SSE) i.e. *quasi*-*R* ^2^ = $1 - SSE_{{\hat{y}}} /SSE_{{\bar{y}}}$. Multicollinearity was measured based on Pearson’s correlation coefficients of $\underline{x}_{n \times k}$matrix, **R**_*k* × *k*_, as det **R**_*k* × *k*_ $\in \left[ {0,1} \right]$ and det **R**_*k* × *k*_ = 1 in case of **R**_*k* × *k*_ = **I**.

Hellinger distance, $H \in \left[ {0,1} \right]$, was used as the measure of divergence between two multinomial probability distributions *p* and *q* with *N* classes as $H = \sqrt {1 - \sum\nolimits_{i = 1}^{N} {\sqrt {p_{i} q_{i} } } }$ (Matusita 1955). Haplotype frequencies were estimated with *maximum likelihood* function (Excoffier and Slatkin 1995). Departure from HWE was measured as $f = \frac{{p_{CC} - p_{C}^{2} }}{{p_{C} \left( {1 - p_{C} } \right)}}$, where *p*_C_ and *p*_CC_ are allele *C* and genotype *CC* frequencies. *f* < 0 in case of deficiency of homozygotes, *f* > 0 corresponds to deficiency of heterozygotes and *f* = 0 when *locus* is in HWE.

As there were no differences in frequencies of tested gene polymorphisms between recurrent miscarriage group (i.e., those with three or more spontaneous abortions) and those with two miscarriages, we could treat both groups as genetically homogenous population. Also, we had no information whether patients with two miscarriages got pregnant later and gave birth to a healthy child. Rather, we could presume that they got miscarriage. So there is no basis to distinguish between Cases A and Cases B group, therefore we pooled these groups in analyses (Tables 1, 2). Our decision to include patients with two miscarriages to analyses was supported also by the fact that many researchers have now included two pregnancy losses to RSA, because childless couples became more prevalent in recent decades (Diejomaoh 2015; Sugiura-Ogasawara et al. 2014).

## Results

The distribution of genotypes of tested polymorphic positions in the women’s group with miscarriages and control women is shown in Table 1. In turn, Table 2 shows the distribution of these SNPs in men from the miscarriage group and from controls.

After analysis of Tables 1 and 2 we could not find evidence for *KIR2DL4*, *LILRB1*, or *HLA*-*G* genotype association with a risk of spontaneous abortion for both women and their partners. However, in this analysis we considered an association of particular polymorphism without including any information of genotype in the remaining SNPs.

Table 3 presents results from the analysis of all tested SNPs and their total phenotype effect with respect to sex. A GT heterozygous woman in position −716 of *HLA*-*G* had over 1.5 times lower chance of miscarriage in comparison to a woman who was homozygous in this SNP (OR = 0.64, *p* = 0.0206). A woman who was heterozygous in the *LILRB1 5651 G*>*A* position, possessed 2.5 times lower probability of abortion in comparison to a homozygous GG or AA woman in this SNP (OR = 0.40, *p* = 0.0131). However, the association of the two discussed SNPs (*HLA*-*G* and *LILRB1*) was not additive. If protective effect of heterozygosity in the *HLA*-*G* –*716 T*>*G* and the *LILRB1 5651 G*>*A* were additive then expected ratio for double heterozygote would be OR = 0.24. Nevertheless, observed value is OR = 0.62 with CI 95 % (0.22; 1.76), so we conclude that true effect is not additive and protective effect of double heterozygosity is the same as the one of the two considered. This overall protective effect is estimated as OR = 0.58, CI 95 % (0.42; 0.81). We can also infer from Table 3 that a man’s genotype in a fragment of polyA of the *KIR2DL4* gene was associated with miscarriage of his partner. The likelihood of abortion in a woman with a 9A/10A partner was 1.49 times higher than in a woman with a homozygous partner (*p* = 0.0288). To summarize the results in Table 3 we can say that all these factors were important in terms of the protection (women’s −716 *HLA*-*G* and *LILRB1*) or susceptibility (men’s *KIR2DL4* 9A/10A) to miscarriage (*p* = 0.00968).

**Table 3:** Estimated odds ratios (OR) in model for odds of miscarriage by *HLA*-*G* (–716), *LILRB1* (rs41308748) and *KIR2DL4* polyA polymorphisms

Polymorphism	OR	CI 95 %		*p*
HLA-G −716 women: GT	0.64	0.43	0.93	0.0206
LILRB1 women: GA	0.40	0.19	0.82	0.0131
KIR2DL4 men: 9A/10A	1.49	1.04	2.13	0.0288
HLA-G −716 women: GT × LILRB1 women: GA	0.62	0.22	1.76	0.0761

det**R**_3×3_ = 0.998; *quasi*-R^2^ = 0.0266

*χ*_df=4_^2^ = 13.35, *p* = 0.00968

*β*_0_ = 0.3955 CI 95 % (0.074; 0.717)

Woman: *HLA*-*G* (–716) *not* GT & *LILRB1* *not* GA plus partner *KIR2DL4* *not* 9A/10A assumed as baseline

*P* probability, *OR* odds ratio, *CI 95* *%* confidence interval

Otherwise, none of the remaining polymorphic positions had any association with miscarriage (*x*_*df*=13_^2^ = 3.02, *p* = 0.9979), including woman’s genotype in polyA of the *KIR2DL4* gene (*p* = 0.8397).

*KIR2DL4* and *LILRB1* genes are parts of the same chromosome and are located in the leukocyte region complex, so we analyzed haplotype frequencies in SNPs of both genes in women and men from patients and the control group (Supplementary Material 3). We found no association of particular haplotype with miscarriage, both for women (*p* = 0.2043) and men (*p* = 0.3804). Although the haplotype 10A-C-A-A-A was 18 times less frequent in cases than in controls, but its frequency was low in both groups, as it contained rare *LILRB1* *5651A* allele.

The analysis of *HLA*-*G* haplotypes (Table 4) presents no differences in all haplotypes frequencies of tested females groups and also their partners, so the Hellinger distances are minor (*H* = 0.075, *H* = 0.066, respectively).

**Table 4:** Haplotype frequencies of *HLA*-*G* polymorphisms in men and women group among miscarriages cases and controls, sorted by frequency in cases women

HLA-G haplotypes			Women			Men		
ins/del	−725	−716	Cases (%)	Controls (%)	RR	Cases (%)	Controls (%)	RR
ins	C	G	37.85	39.00	0.97	46.7	41.78	1.12
del	C	T	33.59	37.66	0.89	30.05	29.22	1.03
del	G	T	15.63	14.55	1.07	13.11	15.30	0.86
del	C	G	10.64	6.66	1.60	8.10	10.50	0.77
ins	T	T	2.02	1.10	1.84	1.77	3.20	0.55
ins	G	T	0.28	0.74	0.38	0.27	0.00	–
Σ			100	99.71	–	100	100	–
Cases vs. controls			*χ*_*df*=5_^2^ = 7.34; *p* = 0.1965 *H* = 0.075			*χ*_*df*=5_^2^ = 5.68; *p* = 0.3386 *H* = 0.066		

*Ins* 14 bp insertion in 3′UTR of *HLA*-*G*, *Del* 14 bp deletion in 3′UTR of *HLA*-*G*, *RR* ratio Cases/Controls, *P* probability, *H* Hellinger distance

The final step of our analysis was to include some clinical information concerning the patient group (age, number of miscarriages, week of miscarriage, autoantibodies, antiphospholipid syndrome, *MTHFR 677C*>*T*, *MTHFR 1298A*>*C*). None of the above-cited variables exhibited an association with the number of miscarriages (*χ*_*df*=11_ = 5.48, *p* = 0.9055).

## Discussion

In our case–control study, we tried to elucidate an association of particular genetic variants of *KIR2DL4*, *LILRB1* and its ligand *HLA*-*G* in women as well as in their partners with susceptibility to spontaneous abortion. To date, no association of maternal *KIR2DL4* polymorphism with RSA and preeclampsia has been reported (Witt et al. 2002, 2004), and our results are concordant with Witt et al. (2004). Moreover, reports of fertile women who lacked *KIR2DL4* and delivered babies have been published by Gómez-Lozano et al. (2003) and Nowak et al. (2011) implying that this gene is not essential for reproduction. However, *KIR2DL4* is one of the “framework” genes, and its loss is rare because only several individuals lacking this gene have been reported to date (Gómez-Lozano et al. 2003; Niepiekło-Miniewska et al. 2014; Nowak et al. 2011). On the other hand, a lack of *KIR2DL4* gene and its receptor on the surface of immune cells might be compensated by the presence of receptors belonging to the LILR family, e.g. LILRB1. Indeed, we found that the women’s GA heterozygosity in *LILRB1* seems protective (Table 3). It is worth emphasizing that the distribution of *LILRB1* genotypes in the control group is in HWE, but in the group of patients is not (Table 1). This independently suggests the association of *LILRB1* GA with protection against disease. rs41308748, as the splicing site, could have an influence on the creation of LILRB1 variants with decreased inhibitory function of receptors resulting in activation of local immune cells to produce cytokines and growth factors necessary for successful embryo implantation and subsequent maintenance of pregnancy. Moreover, one of the ITIMs in the LILRB1 was reported to possess an immunoreceptor tyrosine-based switch motif (ITSM; SXVXXV), and the binding of adaptors signaling lymphocyte activation molecule-associated protein and Ewing’s sarcoma-associated transcript to an ITSM can convert receptors from inhibitory to activating function (Li et al. 2009). Such isoforms may act instead of KIR2DL4, which emerged in this study not associated with susceptibility to miscarriage or protection against disease in female patients. Moreover, the studies on the crystal structure of KIR2DL4 showed that this receptor oligomerizes to tetramers possibly because of the absence of D0 domain glycosylation (Moradi et al. 2015). This was supposed to preclude an interaction of receptor with HLA. Indeed, Moradi et al. (2015) were unable to detect an interaction between KIR2DL4 and a panel of 100 pHLA-Ia or HLA-G by single HLA-antigen bead assay and by surface plasmon resonance. Therefore, a self-association of KIR2DL4 could regulate ligand binding and subsequent signal transduction.

An interesting aspect of our analysis was the observation of the significantly higher frequency of 9A/10A *KIR2DL4* genotype in men belonging to the miscarriage group (Table 3). Why the genotype of man’s *KIR2DL4* is important in susceptibility to miscarriage of his partner, but *KIR2DL4* genotype of the woman is not, is hard to explain. There are no literature data regarding the role of *KIR2DL4* genotype of a man in miscarriage of his partner. Recently, the expression of some KIRs (2DL1, 2DL2/3, 3DL1 but not KIR2DL4 which was not tested in that study) in neonatal cord blood has been reported by Schonberg et al. (2011), showing that KIR repertoires of neonatal NK cells are diverse but not biased toward recognition of self HLA class I. However, neonatal NK cells were functional at the level of antibody-dependent cellular cytotoxicity and cytokine production. As early as 1992, Phillips et al. revealed that human NK cells develop early in utero, as they have been detected in fetal liver at the sixth week of gestation and in fetal spleen at gestational week 15. The evidence for the NK cells differentiation and inhibitory KIR expression was also identified by Ivarsson et al. (2013) in the majority of fetal lung NK cells but also spleen and bone marrow from 15 to 22 gestational age. These fetal NK cells were hyporesponsive to HLA-negative target cells. From tenth week of gestation on, the fetus absorbs amniotic fluid which contains cytokines, antibodies, maternal cells and sometimes pathogens. High proportion of differentiated NK cells in the fetal lungs was supposed to protect against fetal infection. In turn, the hyporesponsiveness of these cells to HLA-negative cells could potentially prevent the recognition of maternal semi-allogeneic cells by fetal NK cells. Whether scenario, in which the paternal *KIR2DL4* allele inherited by the fetus could have an impact on NK cells responses in the developing fetus is likely, remains to be elucidated. However, the expression of KIR2DL4 and LILRB1 receptors in both primary trophoblasts and trophoblastic cell lines (JAr and JEG-3) has been described by Guo et al. (2013). Moreover, these receptors were functional as trophoblast invasion was induced by binding soluble HLA-G5 to KIR2DL4 and LILRB1. In addition, KIR2DL4 can interact with heparan sulfate/heparin glycosaminoglycans (GAGs), an alternative ligand, and these interactions can affect receptor function (Brusilovsky et al. 2013, 2014). Therefore, we may hypothesize that trophoblast KIR2DL4 inherited from the father may interact with GAG-containing proteoglycans, and that this interaction may be affected by KIR2DL4 polymorphism.

All genotype distributions in control women were in HWE (Table 1). Moreover, frequencies of 9A and 10A position 9620 insertion/deletion alleles and alleles in the two intronic positions 9571 and 9797 of *KIR2DL4* approximated to 50 %. The presence of 9A and 10A alleles in populations in equal frequency has been postulated to indicate balancing selection (Goris et al. 2009; Le Page et al. 2013; Witt et al. 2000). We previously reported that above-mentioned *KIR2DL4* polymorphisms in healthy fertile Polish population were in strong positive linkage disequilibrium (Nowak et al. 2015). This complete LD between *KIR2DL4* 9620 and 9571 positions allowed us to confirm our results in two independent methods, namely HRM and RFLP.

Polymorphisms of *HLA*-*G* in promoter positions −716 T>G and −725 G>C>T were previously reported to be linked with methylation status of the gene which had an influence on the transcriptional activity, because of the vicinity to the interferon-stimulated response element (Donadi et al. 2011). The −725G variant present in both spouses was associated with sporadic miscarriage in Hutterites (a genetically isolated sect of Anabaptists) (Ober et al. 2003). We also tested possible association of the −725G allele with the number of miscarriages. However, we did not find such an association in our outbred group of sporadic or recurrent abortion, neither in women nor in their partners, separately or in couples. On the other hand, we found a protective effect from miscarriage of −716 GT heterozygosity. Possibly, the higher heterozygosity in this locus may be enforced by natural selection as it has been observed for other MHC genes (Meyer et al. 2006; Penn et al. 2002). Heterozygotes in MHC may perceive twice more antigens than homozygotic carriers so they could be resistant to broader spectrum of pathogens. Therefore, heterozygosity in HLA-G may here play a role of a marker of classical HLA heterozygosity in mothers, which might favor a maintenance of pregnancy. Moreover, mother could transmit her HLA-G allele to the fetus which, when expressed in the fetus, could be recognized by her LILRB1. This may result in secretion of cytokines from decidual leucocytes to induce immune tolerance allowing trophoblast migration and vascular remodeling during placental development.

Deviation from HWE in the 14 bp in/del of *HLA*-*G* and SNP in *LILRB1* not only in male partners from the miscarriage group but also in control men was seen. Note that control men were selected from the whole Polish population by their partners having at least two healthy-born children and no history of abortion. Consequently, this group was not random as in the case of a group of, e.g. unrelated blood donors and therefore a bias in our study could occur. Indeed, in our earlier study (Wiśniewski et al. 2015) a larger control group, unselected for fertility, did not deviate from HWE in these both genes. However, we feel that our 219 fertile couples are better control for miscarriage and other pregnancy disorders, than those published earlier by others, e.g. primiparous women with normal pregnancies and, where available, their male partners (Hiby et al. 2008, 2010). Some studies included too small group of healthy couples (Ozturk et al. 2012; Vargas et al. 2009; Varla-Leftherioti et al. 2005), or both controls and cases were of mixed ethnicity (Faridi et al. 2009). Then, both the patients and controls have to be carefully selected on the basis of their clinical characteristics, age and ethnicity. We believe that our control and cases groups meet all these criteria.

In summary, our results suggest that a woman’s heterozygosity in *HLA*-*G* −716 T>G and the *LILRB1* 5651 G>A might be advantageous for success of reproduction, but the partner’s heterozygosity in 9A/10A *KIR2DL4* alleles might not.

## Electronic supplementary material

Below is the link to the electronic supplementary material.

Supplementary Fig. 1 Electrophoregram of *MTHFR 677C* > *T* genotyping. M: marker O’GeneRuler (Thermo Scientific), N: not digested, CC: C homozygote, CT: heterozygote, TT– T homozygote (JPEG 18 kb)

Supplementary Fig. 2 Sequencing of 245 bp PCR product to detect *MTHFR 677C* > *T* polymorphism (JPEG 29 kb)

Supplementary Fig. 3 Electrophoregram of *MTHFR 1298A* > *C* genotyping, M: marker O’GeneRuler (Thermo Scientific), N: not digested, CC: C homozygote, AC: heterozygote, AA: A homozygote (JPEG 18 kb)

Supplementary Fig. 4 Sequencing of 276 bp PCR product to detect *MTHFR 1298A* > *C* polymorphism (JPEG 36 kb)

Supplementary Fig. 5 Electrophoregram of *LILRB1 5671G* > *A* genotyping. M: marker O’GeneRuler (Thermo Scientific), N: not digested, GG: G homozygote, GA: heterozygote, AA: A homozygote (JPEG 16 kb)

Supplementary Fig. 6 Sequencing of 233 bp PCR product to detect *LILRB1 5671G* > *A* polymorphism (JPEG 33 kb)

Supplementary material 7 (DOC 111 kb)

Supplementary material 8 (DOC 72 kb)

Supplementary material 9 (DOC 195 kb)
