# Supplementary figures and images for: Possible Role of HLA-G, LILRB1 and KIR2DL4 Gene Polymorphisms in Spontaneous Miscarriage
Source: Arch Immunol Ther Exp (Warsz). 2016 Mar 14;64(6):505–14. doi: 10.1007/s00005-016-0389-7 (PMC5085992; doi:10.1007/s00005-016-0389-7)

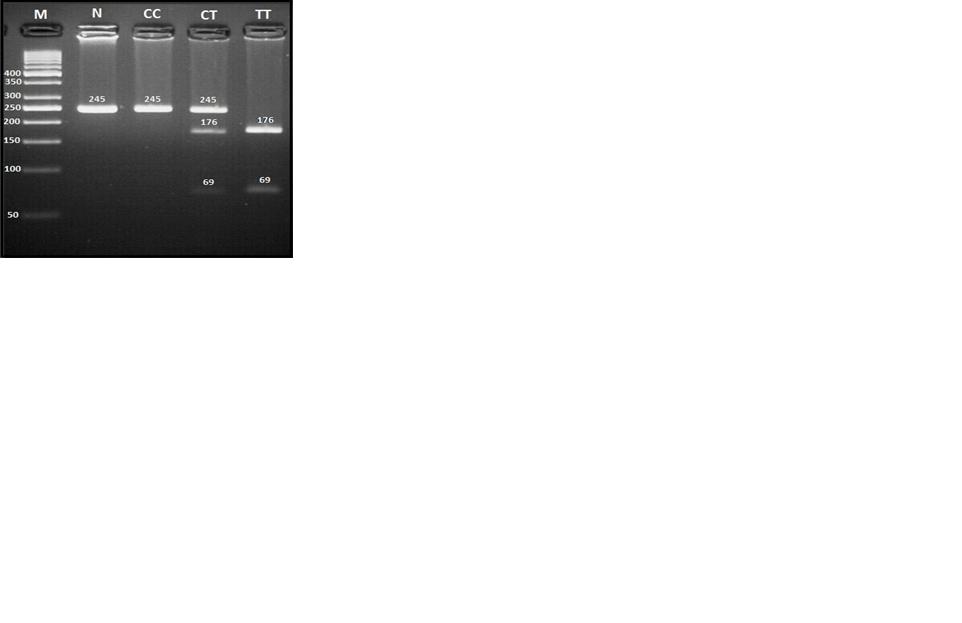

Supplement: Supplementary file 1 — Supplementary Fig. 1 Electrophoregram of MTHFR 677C > T genotyping. M: marker O’GeneRuler (Thermo Scientific), N: not digested, CC: C homozygote, CT: heterozygote, TT– T homozygote (JPEG 18 kb) [file 5_2016_389_MOESM1_ESM.jpg]

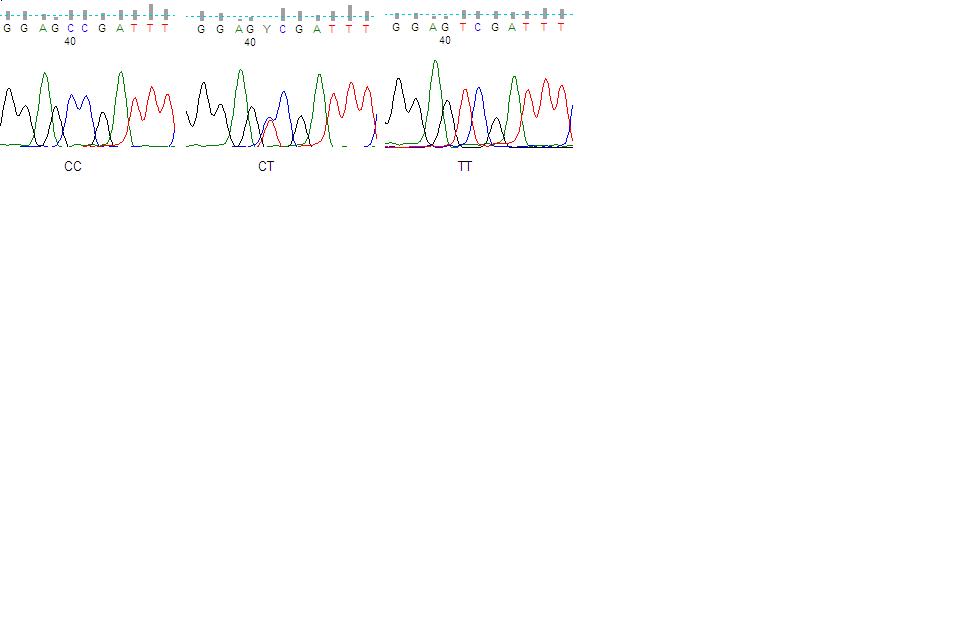

Supplement: Supplementary file 2 — Supplementary Fig. 2 Sequencing of 245 bp PCR product to detect MTHFR 677C > T polymorphism (JPEG 29 kb) [file 5_2016_389_MOESM2_ESM.jpg]

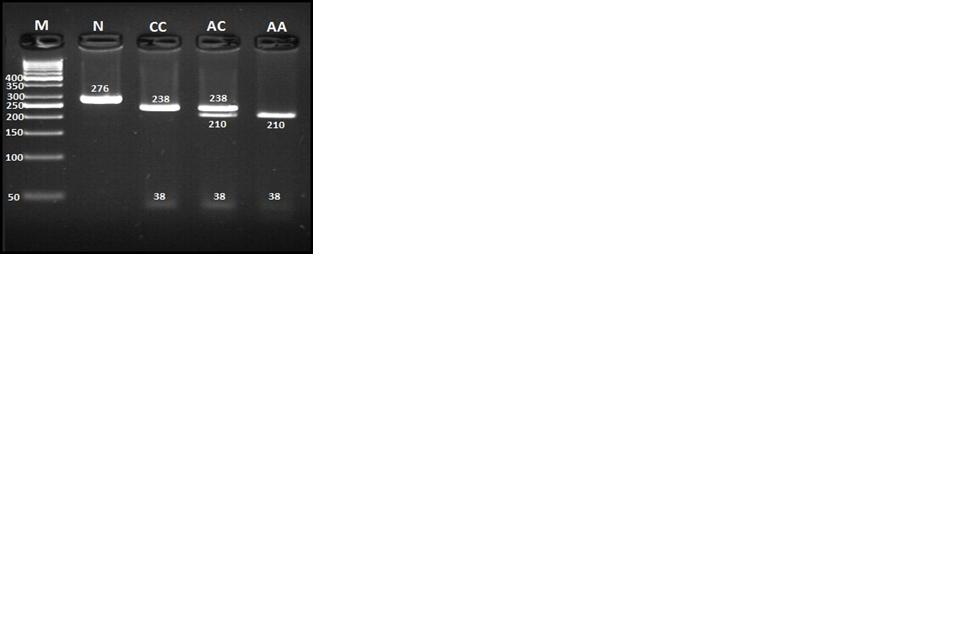

Supplement: Supplementary file 3 — Supplementary Fig. 3 Electrophoregram of MTHFR 1298A > C genotyping, M: marker O’GeneRuler (Thermo Scientific), N: not digested, CC: C homozygote, AC: heterozygote, AA: A homozygote (JPEG 18 kb) [file 5_2016_389_MOESM3_ESM.jpg]

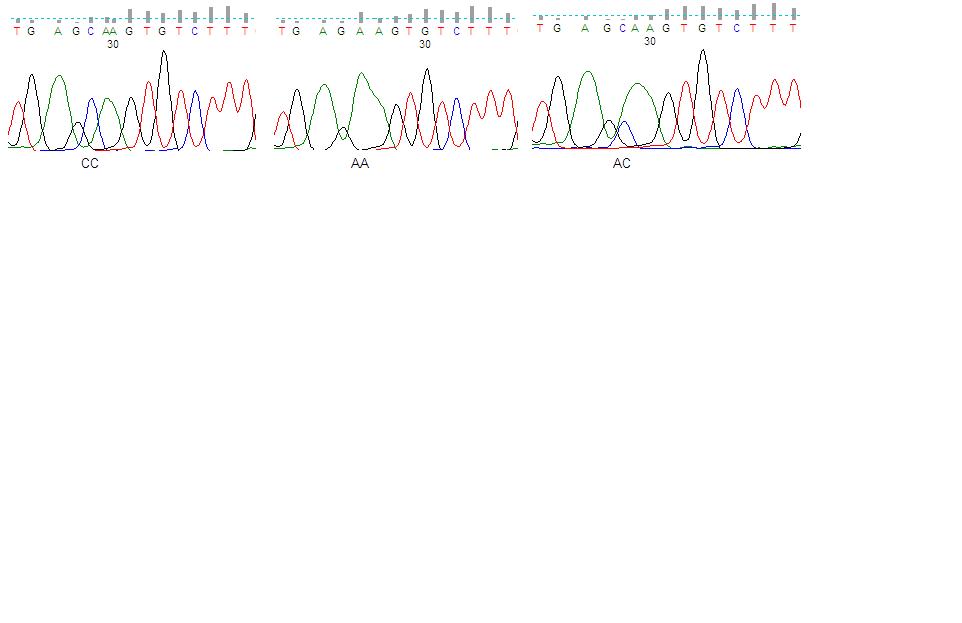

Supplement: Supplementary file 4 — Supplementary Fig. 4 Sequencing of 276 bp PCR product to detect MTHFR 1298A > C polymorphism (JPEG 36 kb) [file 5_2016_389_MOESM4_ESM.jpg]

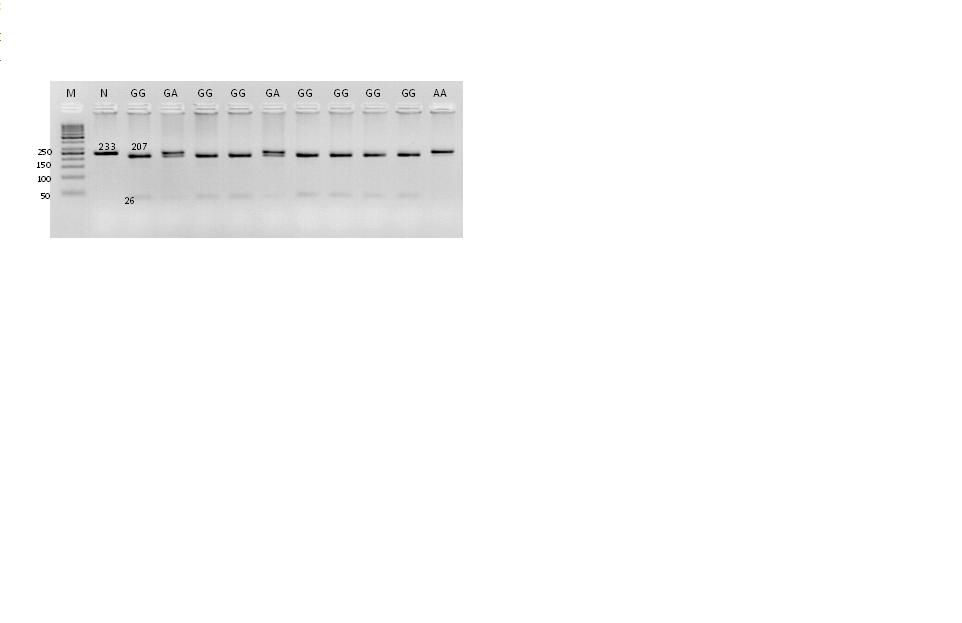

Supplement: Supplementary file 5 — Supplementary Fig. 5 Electrophoregram of LILRB1 5671G > A genotyping. M: marker O’GeneRuler (Thermo Scientific), N: not digested, GG: G homozygote, GA: heterozygote, AA: A homozygote (JPEG 16 kb) [file 5_2016_389_MOESM5_ESM.jpg]

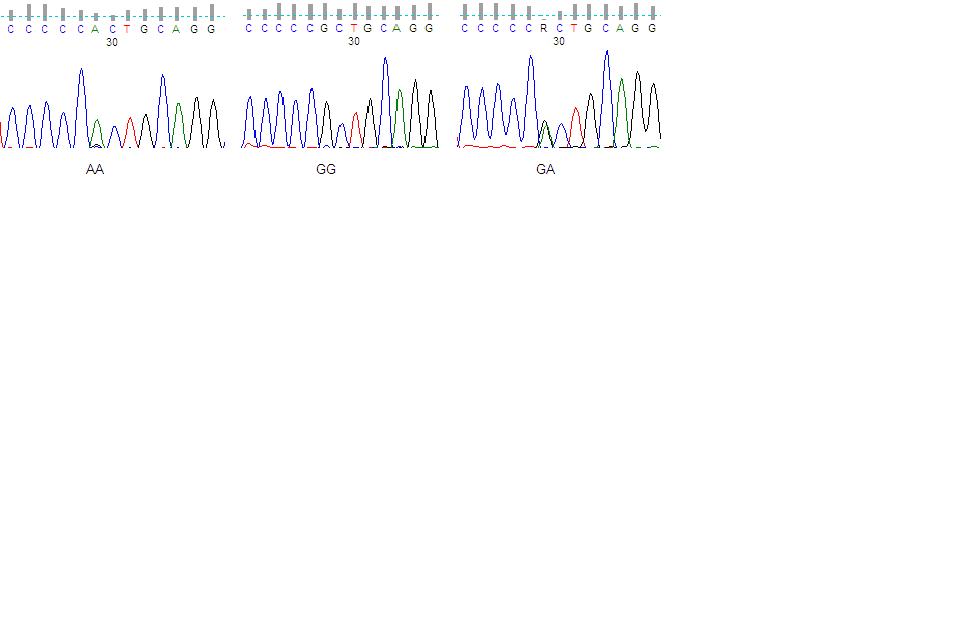

Supplement: Supplementary file 6 — Supplementary Fig. 6 Sequencing of 233 bp PCR product to detect LILRB1 5671G > A polymorphism (JPEG 33 kb) [file 5_2016_389_MOESM6_ESM.jpg]
