# Supplementary material for: Possible Role of HLA-G, LILRB1 and KIR2DL4 Gene Polymorphisms in Spontaneous Miscarriage
Source: Arch Immunol Ther Exp (Warsz). 2016 Mar 14;64(6):505–14. doi: 10.1007/s00005-016-0389-7 (PMC5085992; doi:10.1007/s00005-016-0389-7)
Supplement: Supplementary file 7 — Supplementary material 7 (DOC 111 kb) [file 5_2016_389_MOESM7_ESM.doc]

Possible role of *HLA-G*, *LILRB1* and *KIR2DL4* gene polymorphisms in spontaneous miscarriage

Archivum Immunologiae et Therapiae Experimentalis

Izabela Nowak, Andrzej Malinowski, Ewa Barcz, Jacek R. Wilczyński, Marta Wagner, Edyta Majorczyk, Hanna Motak-Pochrzęst, Małgorzata Banasik, Piotr Kuśnierczyk

**Corresponding authors**: Izabela Nowak, izan@iitd.pan.wroc.pl, and Piotr Kuśnierczyk, pkusnier@iitd.pan.wroc.pl, Laboratory of Immunogenetics and Tissue Immunology, Ludwik Hirszfeld Institute of Immunology and Experimental Therapy, Polish Academy of Sciences, Rudolfa Weigla 12, 53-114 Wrocław, Poland

**Supplementary material 1** Primer sequences for *MTHFR* *677C>T* and *MTHFR* *1298A>C* polymorphism genotyping

| **Polymorphism** | **Primer** | **Primer sequences**  **5’ → 3’** | **Amplicon (bp)** |
| --- | --- | --- | --- |
| *MTHFR* *677C>T* | Forward | CAAAGGCCACCCCGAAGC | 245 |
|  | Reverse | AGGACGGTGCGGTGAGAGTG |  |
| *MTHFR 1298A>C* | Forward | GCAAGTCCCCCAAGGAGG | 276 |
|  | Reverse | GTGGAGGTCTCCCAACTTAC |  |

For *MTHFR 677C>T* genotyping the reaction mixture contained: 1.0 µl of genomic DNA (100 ng/µl), 1.0 µl of 10× buffer, 0.6 µl of MgCl_2_ (25 mM), 0.4 µl of deoxynucleotides (10 mM), 0.3 µl of forward primer (5 µM), 0.3 µl of reverse primer (5 µM), 0.08 µl of Taq polymerase (5 U/µl) and 6.32 µl of deionized water. Polymerase chain reaction was carried out as follows: 95°C for 3.0 min, (95°C – 30 s, 65.5°C – 30 s, 72°C – 35 s) ×34 and 72°C for 10.0 min. Enzyme digestion was conducted at 37°C for 3 h with 2 U of HinfI (New England Biolabs) per sample. Products of digestions were electrophoresed on 3% agarose. We detected respective bands for TT – 176 and 69 base pairs (bps); CT – 245, 176, 69 bps; CC – 245 bps (**Supplemental Fig. 1** and **2**).

For *MTHFR 1298A>C* genotyping the reaction mixture differed from the mixture described above only in primers. Polymerase chain reaction was carried out as follows: 95°C for 3.0 min, (95°C – 30 s, 56°C – 30 s, 72°C – 35 s) ×34 and 72°C for 10.0 min. Enzyme digestion was conducted at 37°C for 1 h with 2 U of MboII (New England Biolabs) per sample. Products of digestions were electrophoresed on 3% agarose. We detected respective bands for CC – 238, 38 bps; CA – 238, 210 bps; AA – 210, 38, 28 bps (**Supplemental Fig. 3** and **4**)
