# Supplementary material for: Possible Role of HLA-G, LILRB1 and KIR2DL4 Gene Polymorphisms in Spontaneous Miscarriage
Source: Arch Immunol Ther Exp (Warsz). 2016 Mar 14;64(6):505–14. doi: 10.1007/s00005-016-0389-7 (PMC5085992; doi:10.1007/s00005-016-0389-7)
Supplement: Supplementary file 8 — Supplementary material 8 (DOC 72 kb) [file 5_2016_389_MOESM8_ESM.doc]

Possible role of *HLA-G*, *LILRB1* and *KIR2DL4* gene polymorphisms in spontaneous miscarriage

Archivum Immunologiae et Therapiae Experimentalis

Izabela Nowak, Andrzej Malinowski, Ewa Barcz, Jacek R. Wilczyński, Marta Wagner, Edyta Majorczyk, Hanna Motak-Pochrzęst, Małgorzata Banasik, Piotr Kuśnierczyk

Corresponding authors: Izabela Nowak, izan@iitd.pan.wroc.pl, and Piotr Kuśnierczyk, pkusnier@iitd.pan.wroc.pl, Laboratory of Immunogenetics and Tissue Immunology, Ludwik Hirszfeld Institute of Immunology and Experimental Therapy, Polish Academy of Sciences, Rudolfa Weigla 12, 53-114 Wrocław, Poland

**Supplementary material 2** Primer sequences for *LILRB1* *5651G>A* polymorphism genotyping

| Polymorphism | Primer | Primer sequences  5’ → 3’ | Amplicon (bp) |
| --- | --- | --- | --- |
| *LILRB1* | Forward | CACGTTCCTTCCCTCTCACT | 233 |
|  | Reverse | CTCCCATGCATTCCAGACT |  |

The reaction mixture contained 1.0 μl of PCR buffer, 0.3 μl of primers (5 μM), 1.0 μl of DNA (100 ng/μl), 0.6 μl of MgCl2 (25 mM), 0.4 μl of deoxynucleotide (10 mM), 0.08 μl of Taq polymerase (Fermentas) and 6.32 μl of deionized water. Polymerase chain reaction was carried out as follows: 94°C for 4 min, then (95°C for 35 s, 57°C for 30 s, 72°C for 35 s) x 30 cycles. Enzyme digestion was conducted at 37°C for 3 hours with 2 U of Aci I (Fermentas) per sample. Digestion products were electrophoresed on 3% agarose. We detected respective bands for AA – 233; GA – 233, 207, 26 and GG – 207, 26 base pairs (**Supplemental Fig. 5**). Reference samples for this SNP were sequenced in the reverse direction (**Supplemental Fig. 6**).
