# Supplementary material for: Possible Role of HLA-G, LILRB1 and KIR2DL4 Gene Polymorphisms in Spontaneous Miscarriage
Source: Arch Immunol Ther Exp (Warsz). 2016 Mar 14;64(6):505–14. doi: 10.1007/s00005-016-0389-7 (PMC5085992; doi:10.1007/s00005-016-0389-7)
Supplement: Supplementary file 9 — Supplementary material 9 (DOC 195 kb) [file 5_2016_389_MOESM9_ESM.doc]

Possible role of HLA-G, LILRB1 and KIR2DL4 gene polymorphisms in spontaneous miscarriage

Archivum Immunologiae et Therapiae Experimentalis

Izabela Nowak, Andrzej Malinowski, Ewa Barcz, Jacek R. Wilczyński, Marta Wagner, Edyta Majorczyk, Hanna Motak-Pochrzęst, Małgorzata Banasik, Piotr Kuśnierczyk

Corresponding authors: Izabela Nowak, izan@iitd.pan.wroc.pl, and Piotr Kuśnierczyk, pkusnier@iitd.pan.wroc.pl, Laboratory of Immunogenetics and Tissue Immunology, Ludwik Hirszfeld Institute of Immunology and Experimental Therapy, Polish Academy of Sciences, Rudolfa Weigla 12, 53-114 Wrocław, Poland

**Supplementary Table 1.** Haplotype frequencies of *KIR2DL4* and *LILRB1* polymorphisms in men and women groups among miscarriage cases and controls, sorted by frequency in case of women

| **Haplotypes** | | | | | **Women** | | | **Men** | | |
| --- | --- | --- | --- | --- | --- | --- | --- | --- | --- | --- |
| KIR2DL4  9A/10A | KIR2DL4  9571 | KIR2DL4  9769 | KIR2DL4  9797 | LILRB1  5651 | Cases (%) | Controls (%) | RR | Cases (%) | Controls (%) | RR |
| 9A | T | C | G | G | 46.94 | 49.36 | 0.95 | 48.99 | 49.66 | 0.99 |
| 10A | C | C | A | G | 28.68 | 23.41 | 1.23 | 24.73 | 27.51 | 0.90 |
| 10A | C | A | A | G | 15.29 | 15.81 | 0.97 | 14.33 | 12.33 | 1.16 |
| 9A | T | C | G | A | 4.33 | 3.84 | 1.13 | 6.06 | 5.14 | 1.18 |
| 10A | C | C | A | A | 2.63 | 2.84 | 0.93 | 3.55 | 1.71 | 2.08 |
| 10A | T | C | G | G | 1.52 | 2.06 | 0.74 | 0.00 | 1.60 | 0.00 |
| 10A | C | A | A | A | 0.11 | 2.00 | 0.06 | 1.33 | 0.46 | 2.89 |
| Σ | | | | | 99.5 | 99.32 | - | 98.99 | 98.41 | - |
| Cases vs. Controls | | | | | χ^2^_df=6_ = 8.49; p = 0.2043  H = 0.135 | | | χ^2^_df=5_= 5.3; p = 0.3804  H = 0.157 | | |

*P* probability, *RR* ratio cases/controls, *H* Hellinger distance
